# Co-Administration of Drugs and Parenteral Nutrition: In Vitro Compatibility Studies of Loop Diuretics for Safer Clinical Practice

**DOI:** 10.3390/pharmaceutics12111092

**Published:** 2020-11-13

**Authors:** Szymon Tomczak, Maciej Stawny, Anna Jelińska

**Affiliations:** Department of Pharmaceutical Chemistry, Poznan University of Medical Sciences, 6 Grunwaldzka, 60-780 Poznań, Poland; mstawny@ump.edu.pl (M.S.); ajelinsk@ump.edu.pl (A.J.)

**Keywords:** drug interactions, parenteral nutrition, Y-site administration, loop diuretics, compatibility

## Abstract

Parenteral nutrition (PN) admixtures are prone to interacting with drugs administered intravenously via a common catheter. This may cause a threat to a patient’s health and life. The literature that has been reported on the compatibility of loop diuretics with PN presents conflicting results. This work aimed to study the compatibility of furosemide and torsemide with PN used in clinical practice. Undiluted solutions of drugs were mixed with PN at various ratios determined by flow rates. In order to assess compatibility, visual control was followed by pH measurement, osmolality, mean emulsion droplet diameter (MDD), and zeta potential upon mixing and at 4 h of storage. No macroscopic changes that indicated lipid emulsion degradation were observed. After the addition of the drugs, the value of pH ranged from 6.37 ± 0.01 to 7.38 ± 0.01. The zeta potential was in reverse proportion to the drug concentration. The addition of the drugs did not affect the MDD. It may be suggested that the co-administration of furosemide or torsemide and PN caused no interaction. The absence of such signs of unwanted interactions allowed for the co-administration of the mentioned loop diuretics and PN at each of the studied ratios.

## 1. Introduction

Critically ill patients require special care due to their life-threatening conditions, diseases, and malnutrition. The incidence of malnutrition worsens over time in patients who require prolonged hospitalization [1,2]. The pathophysiology of malnutrition in intensive care unit (ICU) patients is complex. Catabolic reactions are caused by hormone secretion (i.e., glucagon, cortisol, catecholamines) and pro-inflammatory cytokines. As a consequence, lipo- and proteolysis, as well as muscle loss and body mass reduction, have been observed to lead to increased morbidity and mortality [3,4]. Nutrition of critically ill patients is an indispensable element of holistic intensive care. The latest ESPEN guidelines [5,6] indicate that clinical nutrition should be considered for each patient remaining in the intensive care unit for more than 48 h. In the case of contraindications to oral food intake and enteral nutrition, parenteral nutrition (PN) should be implemented within 3–7 days. The recommended dose of amino acid is 1.3 g per kg bodyweight. The use of immunomodulatory nutrition in intensive therapy is debatable, but it seems that the advantages of this type of preparation outweigh the risk of their use [7].

One of the challenges facing therapeutic teams is the need to provide suitable therapy for patients who are able to receive only parenteral medication. Clinical practice involves the provision of drugs and parenteral nutrition (PN) by means of the Y-site where two or more drugs administered separately are mixed in one catheter [8,9]. This is possible only when the drugs to be provided simultaneously are compatible and avoid liver damage, eye retina blood vessel damage, cardiac arrest, or pulmonary embolism, all of which can have lethal consequences [10,11,12,13]. In addition to drug incompatibility, other intravenous medication administration errors [14,15] may affect a patient’s condition, including the risk of death and the increased cost of medical care resulting from a prolonged stay at the hospital [16,17,18]. In order to reduce the risks involved in PN, the production of PN admixtures and their prescription have been standardized. As a consequence, the number of medical errors has decreased due to their prevention in the preparation process at the hospital pharmacy [14,15,16,19,20,21]. Regarding the economic aspect alone, savings owing to a decline in medical errors have been estimated for ICUs at EUR 26–40 (USD 28–44) per patient per day [19].

A group of patients receiving PN who need particular consideration are those with chronic heart failure, since they require administration of loop diuretics. It has been observed that a continuous infusion of furosemide and torsemide reduces drug resistance and the number of unwanted effects, and improves the patient’s response to treatment, as well as their overall condition [22,23,24,25,26,27].

Regarding the compatibility of furosemide and PN admixtures, Staven et al. [28] demonstrated that the addition of furosemide to a PN admixture results in a precipitate, which disqualifies the admixture as a parenteral drug. In contrast, Bouchoud et al. [29] and Trissel et al. [30] reported no evidence indicating the incompatibility of furosemide administered via a Y-site with a PN admixture. As conflicting research outcomes necessitated further investigation, the problem was addressed in a study on the compatibility of furosemide and torsemide with PN admixtures for the treatment of chronic kidney disease (CKD) [31]. Klek et al. [31] used a different amino acid composition, namely acetylcysteine-containing amino acids dedicated for patients with kidney failure and concluded that furosemide and torsemide were compatible with the PN admixtures at all concentrations applied. This subtle change in the amino acid composition could have affected the drug–PN compatibility. It seems justified to conduct further research to establish the compatibility of loop diuretics with PN admixtures for a wider spectrum of patients.

The aim of our study was to investigate the possibility of interactions following the addition of undiluted furosemide and torsemide solution to PN admixtures that contain basic amino acids, which are administered to the majority of patients who receive PN. The novelty of our approach was the various content of the tested electrolytes and lipid emulsions in the PN admixtures.

## 2. Materials and Methods

### 2.1. Preparation of PN Admixtures

Twelve different PN admixtures (PN 1–PN 12), differing in composition (Table 1), were prepared under aseptic conditions. Lipofundin MCT/LCT (medium- and long-chain triglycerides) 20% (B. Braun, Melsungen AG, Germany) was the injectable lipid emulsion and Aminoplasmal BB 10% E (B. Braun, Melsungen AG, Germany) was the source of amino acids.

### 2.2. Sample Preparation

The drugs used in the study were furosemide (Furosemid Polpharma 10 mg/mL solution for injection; Polpharma, Starogard Gdański, Poland) and torsemide (Trifas 20 5 mg/mL solution for injection; Menarini International Operations, Luxembourg, Luxembourg).

Before the compatibility study, a titration of a drug solution was carried out. Signs of precipitation (e.g., crystals, haziness, turbidity) were not observed during titration with 0.1 M HCl up to pH responding to pH value of PN admixture.

The maximum flow rate (Fr_max_) of the PN admixture administration was 178.5 mL/h, and the minimum flow rate (Fr_min_) was 104 mL/h. Based on manufacturer data, the flow rate indicated that the furosemide and torsemide should not exceed 24 mL/h and 48 mL/h, respectively. Undiluted drug solutions were used to reach higher concentration, which would represent the worst-case scenario, according to the rule that a higher probability of interaction occurred for higher drug concentrations. In our work, the stability of the drugs was not determined due to the fact that the literature data and the summary of product characteristics confirmed their stability over a period exceeding 4 h.

**Figure 1:**
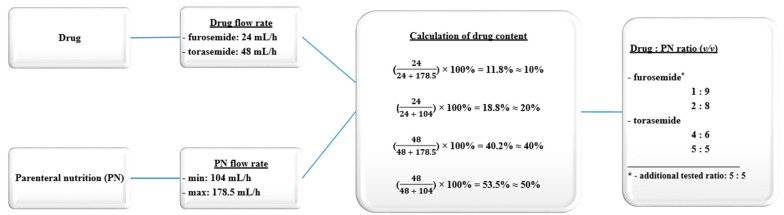
Calculation of the sample preparation.

The extremes of proportions between the drug and the PN were calculated from two infusion rates. Additionally, a sample at a ratio of 5:5 was prepared for comparative reference. For furosemide, 3 ratios were obtained: 1:9, 2:8, and 5:5, where the drug concentrations were 1, 2, and 5 mg/mL, respectively. For torsemide, 2 ratios were obtained: 4:6 and 5:5 where the drug concentrations were 2 and 2.5 mg/mL, respectively. Calculations of sample preparation are detailed in Figure 1. The measurements were taken at two endpoints: upon mixing (t_1_ = 0 h) and after 4 h (t_2_ = 4 h), assuming that most incompatibilities worsen with increasing time. The shortest contact time was represented by the first endpoint (immediately after preparation). The second endpoint (four hours) characterized potential interactions in the case of any variation of infusion time or the volume of the infusion line. The compatibility studies of furosemide and torsemide, as well as 12 different PN admixtures, were based on the measurement of the zeta potential, mean droplet diameter (MDD), osmolality, and pH at the two time points.

### 2.3. Visual Control and Microscopic Inspection

The analysis was conducted by 2 independent observers according to the European Pharmacopeia [32] to detect any evidence of destabilization, such as precipitation, color change, creaming, emulsion separation, or crystallization. The microscopic evaluation, using an optical microscope, was evaluated by two observers. To consider PN admixtures as compatible with furosemide and torsemide, the following acceptance criteria had to be met: practically free from visible particles; no precipitation detected by any observers upon visual inspection; and the absence of macroscopic changes indicating phase separation.

### 2.4. pH Measurement

The pH of the PN admixtures were measured using a Mettler Toledo Seven pH-meter Compact pH/ion S220^®^ (Mettler Toledo, Columbus, OH, USA) for t_1_ and t_2_ at 22 ± 2 °C. Prior to the measurement, the instrument was calibrated at pH 4.00 and 7.00, with the electrode rinsed with distilled water and dried. The measurements were conducted in triplicate for each sample, and the results were expressed as a mean value (X ± SD). To consider PN admixtures as compatible with furosemide and torsemide, the following acceptance criterion had to be met: pH change not exceeding 0.2 after drug addition.

### 2.5. Osmolality Measurement

A 100 µL sample of each PN admixture was transferred to an OSMO-KRIO tube. The osmolality was measured by a freezing point method with a Trident 800LG osmometer (TRIDENT MED, Warsaw, Poland). To consider PN admixtures as compatible with furosemide and torsemide, the following acceptance criterion had to be met: osmolality change not exceeding 5% after drug addition during the evaluation period.

### 2.6. Measurement of Particle Size and Zeta Potential

The determination of particle size and zeta potential was performed according to the methodology described in our previous work [33]. To consider PN admixtures as compatible with furosemide and torsemide, the following acceptance criterion had to be met: MDD must be less than 500 nm with a permissible change less than 10% during the evaluation period [29,34].

## 3. Results

Throughout the experiment, no macroscopic changes indicating lipid emulsion degradation were observed in the PN. Analogously, no evidence of degradation was produced by physicochemical tests. Time-dependent changes in the pH of PN were interpreted as signs of degradation, e.g., lipid hydrolysis with a release of free fatty acids reduced the pH value [35,36,37]. The pH of the PN in the present study remained in the range 6.34–6.72. The lowest values of osmolality were found for PN 3 and PN 9 (low content of amino acids and sodium), 848 and 900 mOsm/kg, respectively. The osmolality of the other PN ranged from 1018 to 1278 mOsm/kg. The size of the lipid emulsion particles is known to be determined mainly by the lipid emulsion source. For all PN, the MDD ranged from 217.4 to 225.1 nm. The zeta potential—i.e., the difference in the potential values of lipid emulsions layers—ranged from −9.8 mV to −17.5 mV. The lowest values were observed for PN 8 and PN 2 (low content of amino acids and magnesium and calcium bivalent cations). The highest zeta potential values were found for PN 10 (high content of lipids, amino acids, and electrolytes).

The pharmaceutical formulation of drugs investigated in this work due to their acidic properties was buffered by alkaline solutions. It has been reported that a large pH difference between drugs or drugs and PN may induce interaction upon mixing [8,28,29]. The pH and osmolality of the furosemide solution were 9.50 ± 0.02 and 295 ± 4 mOsm/kg, respectively. For the torsemide solution, the pH and osmolality were 9.05 ± 0.02 and 461 ± 7 mOsm/kg, respectively.

**Figure 2:**
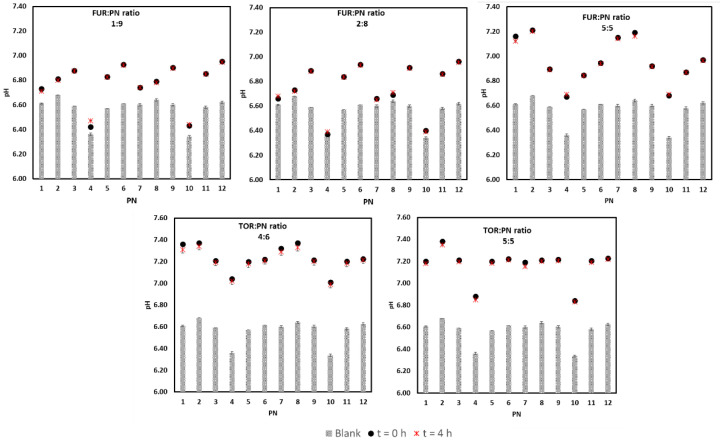
The pH values of drug–PN samples upon preparation and 4 h later (black and red signs), and PN upon preparation (gray bars). The upper part refers to FUR:PN samples in three different ratios, whereas the down part refers to TOR:PN samples in two ratios. FUR, furosemide; PN, parenteral nutrition admixture; TOR, torsemide; Blank, PN without drug.

The addition of the drug solutions did not cause precipitation or color change in the PN at t_1_ and t_2_. The pH of the PN admixture was found to rise after the addition of the drug, reaching a maximum increase of 0.54 in the samples at the ratio 5:5. The pH differences between the PN varied, as shown in Figure 2. After the addition of furosemide, the pH value ranged from 6.37 ± 0.01 (PN 4, ratio: 2:8) to 7.20 ± 0.02 (PN 2, ratio: 5:5). Following the addition of torsemide, the pH value ranged from 6.84 ± 0.01 (PN 10, ratio: 5:5) to 7.38 ± 0.01 (PN 2, ratio: 5:5). The lowest pH values, 6.37−6.47 ± 0.02, were observed for PN 4 (1:9) and PN 10 (2:8).

**Figure 3:**
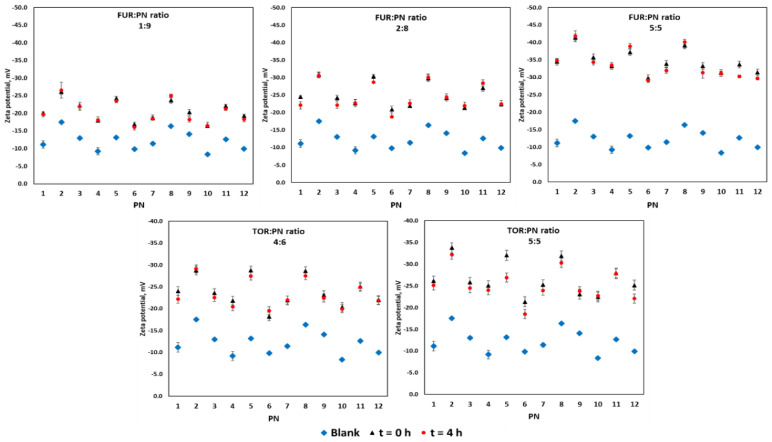
The zeta potential values of drug–PN samples upon preparation and 4 h later (black and red signs), and PN upon preparation (blue signs). The upper part refers to FUR:PN samples in three different ratios, whereas the down part refers to TOR:PN samples in two ratios. FUR, furosemide; PN, parenteral nutrition admixture; TOR, torsemide; Blank, PN without drug.

The zeta potential is known to be determined by the pH values. In this study, the zeta potential was in reverse proportion to the drug concentration in the sample. The lowest zeta potential value, -41.8 mV, was recorded for PN 2 with furosemide at the ratio of 5:5. The highest zeta potential value, −16.0 mV, was observed for PN 6 with furosemide at the ratio of 1:9 (Figure 3).

The addition of drug solutions did not affect the MDD, which ranged from 211 to 231 nm for all PN examined with and without drug content (Table 2). The dynamics of MDD changes did not lead to the formation of a second particle fraction. The changes in the MDD ranged from −1.73 to 2.07%.

**Figure 4:**
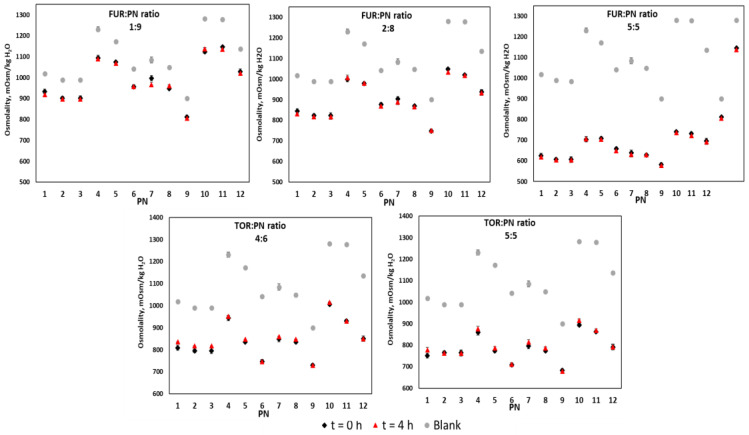
The osmolality of drug–PN samples upon preparation and 4 h later (black and red signs), and PN upon preparation (gray signs). The upper part refers to FUR:PN samples in three different ratios, whereas the down part refers to TOR:PN samples in two ratios. FUR, furosemide; PN, parenteral nutrition admixture; TOR, torsemide; Blank, PN without drug.

As demonstrated in Figure 4, it was found that, as a result of adding low osmolality drug solutions, the osmolality of a given PN admixture decreased. The lowest changes occurred for the furosemide–PN 3 sample at a ratio of 1:9 (Δ = 77 mOsm/kg) and the greatest changes were observed for PN 10 with furosemide at a ratio of 5:5 (Δ = 546 mOsm/kg).

## 4. Discussion

Parenteral administration has been reported to carry the greatest risk of medical error [14,15,16,21]. Particular attention should be directed to PN due to the complexity of the composition of the PN admixture and its two-phase form. Some PN admixtures may contain over 50 individual components that interact with each other in a physical–chemical balance. Any deviation from the protocol designed for the handling of the PN admixture could cause unwanted interactions, such as degradation (lipids hydrolysis or decomposition of amino acids), precipitation (mainly bivalent cations salts), or impaired lipid emulsion (creaming or cracking). In view of such challenges, the therapeutic team faces a highly demanding task that requires extensive expertise. A patient’s health states increase even further when it is therapeutically advisable to combine PN and drug administration. In the absence of data on the safety of co-administration, both solutions must be administered separately [38]. It is therefore necessary to investigate the possibility of interactions following the addition of drugs to PN admixtures.

Loop diuretics such as furosemide and torsemide are common in the management of hypertension and vascular edema. The basicity of these drugs makes them prone to interaction in response to significant pH changes [28,29,31]. The literature data on the compatibility of furosemide and torsemide provide confusing results. This may be explained by the differences in drug concentration and PN admixture composition, especially with regard to what affects pH and buffer capacity [9,28,29,30,31,39,40,41].

Bouchoud et al. [29] investigated the compatibility of 25 drugs with Nutriflex Lipid Special (ready to use, RTU, PN admixture) and found that furosemide was compatible with the admixture at the ratio 1:1. Staven et al. [28] studied 10 drugs for use in children to determine their compatibility with RTU Olimel N5E and Numeta G16E. Incompatibility with the admixture was reported for three drugs, including furosemide, which formed a precipitate upon mixing with Numeta G16E at a pH of 5.5. No signs of incompatibility were observed after adding furosemide to Olimel N5E at a pH above 6.2 for each mixing ratio (1:100, 1:1, and 4:1). Based on the results, the co-administration of furosemide with the pediatric PN admixtures was not recommended. It should be stressed that the pediatric PN admixtures investigated by Staven et al. [28] differed from those studied in our work with regard to composition and physical properties, for instance, pH. It is worth emphasizing that the solubility of furosemide is pH dependent. Furosemide, as an organic acid (pKa = 3.9), is much more soluble at neutral and basic pH values, and it dominates in an anionic form. As previously mentioned, the basicity of the furosemide solution for injection renders it vulnerable to interaction in response to considerable pH changes. An additional experiment was carried out that showed no sign of furosemide precipitating from the solution at pH equaling that of the analyzed PN admixture. Hence, pH should not be considered an exclusive factor responsible for precipitation. Other such factors include the influence of calcium ions and precipitating insoluble calcium salts. It is, therefore, important to examine a wider range of factors in order to assess their potential to affect the physicochemical stability of a given drug–PN admixture combination. The compatibility of torsemide and PN admixtures has not received much attention in the literature, with the only report addressing the combination of PN admixtures with loop diuretics in patients suffering from CKD [31]. The PN admixtures used in that study differed in their amino acid profiles (addition of acetyl cysteine), whereas the loop diuretics were diluted with 0.9% sodium chloride to a concentration range of 0.2–0.9 mg/mL for furosemide and 0.1–1.45 mg/mL for torsemide. The drugs were found compatible with the PN admixtures over a period of 24 h. However, the authors pointed out that further studies were needed for other PN admixture compositions. In our study, we used a wide range of PN admixtures with different amino acid contents, which may have changed the buffer capacity or had an impact on drug–PN compatibilities.

Osmolality refers to the amount of the osmotically active particles in one kilogram of water. Amino acids, carbohydrates (e.g., dextrose and glucose) and electrolytes are significant contributors of solution osmolality. From a clinical point of view, the osmolality of a PN admixture defines the administration route. PN admixtures with osmolality above 900 mOsm/kg should be administered via large veins of the central venous circulation to prevent thrombophlebitis [42]. The lack of changes in both osmolality and pH values confirms their compatibility. With regard to therapeutic safety, changes in pH or osmolality values in PN admixtures may suggest the occurrence of degradation, e.g., lipid hydrolysis with a release of free fatty acids. It has been observed that, below a pH of 5.5, emulsions may be prone to separation, depending on such factors as the electrolyte composition and concentration. [35,36,37]. Regarding visual control, it is not sufficient to rely on the appearance of drug–PN admixture combinations to determine its therapeutic safety. The key parameter that indicates whether or not therapy with a parallel Y-site administration of drugs and PN admixtures poses a threat to a patient’s health is the size of the emulsion particles. Significant variations of its value may reflect the compromised quality of lipid emulsion. [29,34]. The US Pharmacopeia recommends two methods for measuring the emulsion particle size: light scattering (method I) and light or extinction (method II) [34]. In our work, we applied the former method, where the MDD limit was 500 nm. After the addition of drugs (t_1_ and t_2_), changes in the MDD did not exceed 5 nm. All drug–PN admixture combinations satisfied the pharmacopeia requirement, allowing only one fraction of particles with a maximum size of 225.2 ± 3.2 nm (Table 2).

The presence of emulsifiers ensures the stability of the emulsion by inhibiting the connection of individual droplets with each other. Intravenous lipid emulsions may contain soybean or egg yolk lecithin. Negative charges accumulate on the surface of the lipid droplet due to the negatively charged phosphate group of the emulsifier. Repulsive unipolar charges guarantee the durability of the lipid emulsion. Changes in the aqueous phase of the emulsion could disturb this balance and lead to destabilization. The addition of acid solutions, such as glucose or dextrose, affects electronegativity, whereas the addition of cations, especially bi- and tri-valent that neutralize the electric charge, could contribute to reducing the zeta potential and destabilizing the emulsion. On the contrary, the addition of amino acid solutions, due to their buffering properties, prevents charge reduction and degradation of the lipid emulsion [37,42,43].

## 5. Conclusions

The key parameter indicating whether or not therapy with a parallel Y-site administration of drugs and PN admixtures poses a threat to the patient’s health is the size of the emulsion particles. Of the two pharmacopeia methods used to measure emulsion particle size, we used the first. Interactions between drugs and PN admixtures depend on their concentration. Considering the conflicting outcomes of reports on the compatibility of furosemide and PN admixtures, we carried out our experiments using undiluted drug solutions with the advantage of greater interaction detectability. The composition of a PN admixture is one of the factors limiting compatibility studies, since its complexity is responsible for the unpredictability of the results. For this reason, the PN admixtures selected for this study were those commonly used in clinical practice, with a variety of lipids, amino acids, and electrolytes in their contents. This broad spectrum of compositions was aimed at identifying the component instrumental in triggering interactions. Trace elements and vitamins may accelerate the decomposition of drugs; therefore, we attempted to establish compatibility with the PN admixture without these extra compound.

The drug solutions of furosemide and torsemide were found to be compatible with all PN admixtures used in this study. There were no signs that indicated the degradation of lipid emulsions or drugs, which qualifies the said loop diuretics for co-administration with PN admixtures at all studied ratios. These results could form the basis for the development of new guidelines for the use of parenteral medications. The ability to deliver drugs and PN dopants simultaneously has many advantages. It improves patient safety by reducing medical errors, reduces nursing time, and saves money. In addition, administration through one catheter rather than more limits the number of skin perforation and thus potential infections.

In view of the fact that other reports have demonstrated the incompatibility of furosemide, further studies are required to eliminate therapeutic safety risks in this respect.

## Figures and Tables

**Table 1:** Composition of parenteral nutrition admixtures (PNs).

Components	Unit	Low Lipid Content						High Lipid Content					
		PN 1	PN 2	PN 3	PN 4	PN 5	PN 6	PN 7	PN 8	PN 9	PN 10	PN 11	PN 12
Aminoplasmal B 10%E	mL	375.0			875.0			375.0			875.0		
Glucose 40%		625.0											
Lipofundin MCT/LCT 20%		62.5						312.5					
Water for injection		1240.3	1288.9	1341.7	762.5	809.7	848.6	990.3	1038.9	1095.8	512.5	559.7	602.8
Natrium Chloratum 10%		37.5	36.9	0.0	28.2	28.2	0.0	41.3	41.3	0.0	32.5	32.5	0.0
Kalium Chloratum 15%		64.0	64.0	0.0	57.8	57.8	0.0	64.0	64.0	0.0	57.8	57.8	0.0
Calcium Gluconate 10%		44.0	5.6	44.0	44.0	5.6	44.0	44.0	5.6	44.0	44.0	5.6	44.0
Magnesium sulfuricum 20%		11.4	1.9	11.4	9.7	0.4	9.7	11.4	1.9	11.4	9.7	0.4	9.7
Sodium Glycerophasphate		40.3			35.3			36.5			31.5		
Total volume		2500.0	2500.1	2499.9	2500.0	2499.4	2500.1	2500.0	2500.7	2500.3	2500.6	2500.0	2500.6

MCT/LCT: medium- and long-chain triglycerides.

**Table 2:** Mean droplet diameter (MDD) of standard parenteral nutrition with and without furosemide and torsemide.

PN Admixture	MDD ± SD (nm)	FUR:PN Ratio	MDD ± SD (nm)		TOR:PN Ratio	MDD ± SD (nm)	
			PN with FUR			PN with TOR	
			t = 0 h	t = 4 h		t = 0 h	t = 4 h
PN 1	219.7 ± 1.9	1:9	206.9 ± 7.2	209.4 ± 4.5	4:6	211.3 ± 2.8	211.9 ± 5.0
		2:8	206.2 ± 5.5	206.6 ± 5.6			
					5:5	212.0 ± 3.9	211.9 ± 4.5
		5:5	205.2 ± 6.6	202.5 ± 5.4			
PN 2	219.2 ± 1.1	1:9	207.6 ± 10.0	210.6 ± 7.0	4:6	212.5 ± 6.4	216.2 ± 5.1
		2:8	206.7 ± 6.5	207.5 ± 6.6			
					5:5	212.8 ± 4.6	213.5 ± 4.3
		5:5	203.6 ± 6.6	201.3 ± 10.2			
PN 3	217.4 ± 3.9	1:9	210.1 ± 4.9	209.8 ± 4.9	4:6	212.6 ± 3.6	212.1 ± 4.3
		2:8	207.5 ± 6.3	209.0 ± 5.5			
					5:5	212.4 ± 2.8	213.0 ± 3.4
		5:5	207.0 ± 6.4	210.6 ± 7.6			
PN 4	222.0 ± 1.5	1:9	218.5 ± 0.8	219.5 ± 2.6	4:6	223.6 ± 4.4	220.5 ± 3.8
		2:8	217.4 ± 1.9	217.8 ± 2.7			
					5:5	220.3 ± 2.1	221.0 ± 1.6
		5:5	215.6 ± 2.3	215.8 ± 3.5			
PN 5	221.0 ± 1.0	1:9	219.7 ± 2.1	214.4 ± 1.5	4:6	220.9 ± 5.3	216.3 ± 0.9
		2:8	215.9 ± 5.1	213.3 ± 2.3			
					5:5	220.9 ± 4.5	218.9 ± 2.3
		5:5	212.1 ± 4.9	214.5 ± 5.7			
PN 6	221.4 ± 1.1	1:9	224.1 ± 2.2	216.5 ± 1.2	4:6	221.5 ± 3.4	220.9 ± 6.7
		2:8	217.3 ± 4.9	219.7 ± 2.6			
					5:5	222.5 ± 3.2	221.0 ± 1.9
		5:5	213.3 ± 5.1	213.8 ± 6.1			
PN 7	220.0 ± 1.4	1:9	212.0 ± 5.9	211.6 ± 5.0	4:6	215.5 ± 4.3	215.1 ± 4.7
		2:8	209.3 ± 4.7	208.7 ± 5.3			
					5:5	215.9 ± 4.7	214.4 ± 2.8
		5:5	201.4 ± 8.6	203.4 ± 6.0			
PN 8	220.6 ± 1.6	1:9	222.8 ± 2.0	213.3 ± 6.6	4:6	213.0 ± 6.0	215.4 ± 4.4
		2:8	222.4 ± 3.7	211.9 ± 7.5			
					5:5	217.0 ± 3.6	214.7 ± 3.4
		5:5	210.4 ± 5.7	206.7 ± 6.3			
PN 9	219.4 ± 2.6	1:9	213.1 ± 4.5	211.6 ± 3.8	4:6	213.5 ± 6.0	213.2 ± 2.0
		2:8	211.0 ± 6.0	210.1 ± 6.0			
					5:5	214.7 ± 3.0	215.0 ± 2.9
		5:5	205.4 ± 7.7	205.2 ± 6.4			
PN 10	225.1 ± 3.3	1:9	222.8 ± 2.9	224.2 ± 0.5	4:6	225.2 ± 2.4	221.8 ± 5.7
		2:8	221.3 ± 1.7	219.9 ± 1.3			
					5:5	224.5 ± 3.4	220.2 ± 3.2
		5:5	218.5 ± 2.6	216.9 ± 0.3			
PN 11	223.4 ± 0.4	1:9	219.2 ± 1.1	221.8 ± 2.4	4:6	221.1 ± 2.6	220.9 ± 1.6
		2:8	217.9 ± 2.2	217.3 ± 5.0			
					5:5	220.6 ± 2.0	219.7 ± 2.0
		5:5	216.0 ± 4.0	214.0 ± 5.3			
PN 12	220.7 ± 2.0	1:9	218.4 ± 6.7	222.8 ± 3.3	4:6	220.8 ± 2.4	220.3 ± 2.0
		2:8	220.1 ± 3.2	220.0 ± 3.3			
					5:5	220.9 ± 2.8	222.1 ± 1.8
		5:5	218.1 ± 2.1	218.0 ± 1.8			

MDD, mean droplet diameter; PN, parenteral nutrition admixture; SD, standard deviation; t, time; FUR, furosemide; TOR, torsemide.
